# Seroprevalence of Q fever among high-risk occupations in the Ilam province, the west of Iran

**DOI:** 10.1371/journal.pone.0211781

**Published:** 2019-02-19

**Authors:** Ehsan Mostafavi, Leila Molaeipoor, Saber Esmaeili, Ahmad Ghasemi, Maedeh Kamalizad, Manijeh Yousefi Behzadi, Razi Naserifar, Mehdi Rohani, Abdolrazagh Hashemi Shahraki

**Affiliations:** 1 Department of Epidemiology and Biostatistics, Research Centre for Emerging and Reemerging infectious diseases, Pasteur Institute of Iran, Tehran, Iran; 2 National Reference laboratory for diagnosis and research on Plague, Tularemia and Q fever, Research Centre for Emerging and Reemerging infectious diseases, Pasteur Institute of Iran, Akanlu, Kabudar Ahang, Hamadan, Iran; 3 Department of Bacteriology, Faculty of Medical Sciences, Tarbiat Modares University, Tehran, Iran; 4 Department of Parasitology, Faculty of Health, Ilam University of Medical Sciences, Ilam, Iran; 5 Department of Microbiology, Pasteur Institute of Iran, Tehran, Iran; University of Minnesota, UNITED STATES

## Abstract

**Background:**

Q fever is a zoonotic disease of great public health importance in Iran. This disease is presented with high phase I antibody development in chronic and high phase II antibody in the acute form of illness. This study was conducted to evaluate the seroprevalence of Q fever among high-risk occupations in the Ilam province in Western Iran.

**Methods and findings:**

In this cross-sectional study, 367 sera samples were collected from five groups comprised of animal husbandry workers, farmers, butchers, slaughterhouse workers, and park rangers. The collected sera were tested for IgG antibodies against *Coxiella burnetii* using ELISA. The seroprevalence of antibodies against *C*. *burnetii* in phase I and II was 24.38% and 26.37%, respectively (i.e., 32.42% overall). Low educational level, living in rural areas, keeping sheep/goats, ages older than 50 years, and a history of arthropod bites positively correlated with increased risk of Q fever infection. Animal husbandry workers (45.13%) were at higher risk of contracting Q fever compared with other occupations in the study (17.11%).

**Conclusions:**

High seroprevalence of *C*. *burnetii* among high-risk occupations is a serious challenge in the Ilam province. In addition, the high seroprevalence of endemic Q fever in rural and nomadic areas and a higher concentration of occupations who are directly engaged with livestock demonstrate the critical need for preventive medicine education and training in regards to mitigating risk for disease contraction in susceptible groups.

## Introduction

Q fever is a zoonotic disease caused by *Coxiella burnetii* [1] that has been reported from almost all over the world [2]. Domestic ruminants such as cattle, sheep, and goats are the main reservoirs of the disease [3, 4]. Transmission of only a few bacteria is sufficient to cause infection [5]. Humans are primarily infected via the respiratory route after inhaling aerosols and dust particles contaminated with *C*. *burnetii*. Contact with the infected placenta and birth products can have a significant role in the transmission of this agent [5]. Consumption of raw milk and other unpasteurized dairy products, as well as bites of arthropods such as ticks, are less important transmission routes [2, 3]. With its high pathogenicity and resistance to drying and heat, the organism has been modified for the use as a bioweapon. Moreover, it is of significant importance as an agent of bioterrorism [1]. The acute infection in humans is presented after 14 to 39 days of latent phase with nonspecific and variable symptoms ranging from an asymptomatic and self-limiting infection with fatigue, headache, fever, chills, and myalgia to atypical pneumonia or hepatitis. The chronic form of the disease can occur in 1–2% of patients as endocarditis, osteomyelitis, aseptic meningitis, and possibly others [6, 7].

*C*. *burnetii* has two different antigenic phases: phase I and phase II. Such an antigenic difference is important in the diagnosis. In acute cases of Q fever, the titer of antibody against phase II is usually higher than phase I antibody. Acute disease is mostly diagnosed via an increase in the antibody titer within three to four weeks of the onset of the disease. In comparison, in chronic cases, the titer of antibody is higher against phase I compared to phase II. This increase in the titer of antibodies against phases I and II may persist within months to years after the first infection of this disease [8].

Risk factors for Q fever infection in humans include pregnancy, immunosuppression, aneurysm, and cardiac diseases [9]. Although vaccination is recommended for people in high-risk occupations, its usage is not advised to other groups due to the side effects [10–12]. The high prevalence of this disease among older men reflects the occupational risk of this disease [8]. Moreover, previous studies have revealed that exposure to livestock and domestic animals were regarded as a crucial risk factor in the dissemination of Q fever in human societies [13–15]. Animal husbandry workers, farmers, laboratory staff, veterinarians, the park rangers, butchers, and slaughterhouses workers, who are exposed to the reservoirs of the disease, are at higher risk of the infection [1].

The first human cases of acute Q fever in Iran were reported in 1952 in Abadan, the southwest of Iran. Later on, in the following studies, seropositive samples were reported from different parts of the country [16–18]. Several studies conducted over the past six decades in different groups of domestic and wild animals, as well as in human populations, have shown that this disease is endemic in Iran [4, 19–22]. In this regard, a chronic case of Q fever, presenting with endocarditis, has been recently reported in a 72-year-old female in Iran [23]. The recent improvement in the awareness and diagnostic methods caused a significant increase in the number of reports on the disease from Iran’s neighboring countries such as Iraq, Afghanistan, Azerbaijan, Turkey, Saudi Arabia, United Arab Emirates, and Oman [24–29]. Hence, it is crucial to conduct seroepidemiological studies in humans and in animals in order to gain more information about this disease, particularly in the borders of Iran, so as to mitigate the risks of infection. This study was conducted in order to evaluate the seroprevalence of Q fever amongst high-risk populations in the Ilam province, located on the Iran-Iraq border in the west of Iran, in 2015.

## Materials and methods

### Study area

This study was conducted in Ilam province, the west of Iran, in 2015. This province, with a population of about 600,000, is situated in a mountainous region and classified under a Mediterranean climate. The area of Ilam province is 20,000 km^2^; i.e. 1.2% of the whole area of the country.

### Ethics statement

The scientific committee and the medical ethics committee of Pasteur Institute of Iran approved the study. All adult subjects submitted an informed written consent.

### Sampling

In this study, samples were collected using convenience sampling among the different at-risk groups in three cities of Dehloran, Ilam, and Mehran. These three cities are located in the west of Ilam neighboring Iraq. Among all individuals older than 18 years of age, five groups including animal husbandry workers, farmers, butchers, slaughterhouse workers, and park rangers, and those referred to medical diagnostic laboratories for routine testing (as the control group) were included in the study. After obtaining informed consent, the necessary data including the demographic information (i.e., age, gender, educational status, occupation, marital status, city and area of residence), any exposure to risk factors (keeping animals, hunting/consumption of wild animal meat, exposure to ill or dead animals), arthropod bite (tick, flea and mosquito) [30], splashing animal fluids on face/body, consuming non-pasteurized milk or other dairy products, cutting hands or any other parts of body during work, individuals’ awareness, insight and behavior in regards to zoonotic diseases were collected using a researcher-made questionnaire.

About 8 cc of blood sample was obtained from each participant and the samples were immediately transferred to the laboratory. After isolation, the serum samples were kept at -20°C together with the completed questionnaire and kept in a cold chain. Then, all samples were sent to the Epidemiology Department and the Research Center of the Emerging and Reemerging Diseases (the national referral laboratory for plague, tularemia, and Q fever) of Pasteur Institute of Iran.

### Serology

Detecting IgG phases I and II antibodies of *C*. *burnetii*: the collected sera were separately tested for antibodies of phases I and II via ELISA (Serion/Verion Company, Germany) according to the company’s manual. The retrieved optical densities (ODs) were evaluated according to the protocols of Serion/Verion and the IgG phase II was reported quantitatively. The titer of IgG phase II antibody was calculated using a logistic-log model calculation in U/ml. Titers higher than 30 U/ml were considered as positive and those between 20 and 30 and lower than 20 were regarded as borderline and negative, respectively. The definition of the borderline sera was done based on the application guideline of Serion/Verion ELISA kit. In addition, IgG phase I antibody was reported qualitatively. The following formula was applied to determine the upper and lower cut-offs: Lower Cut-off OD: 0.9 × MW (STD), Upper Cut-off OD: 1.1 × MW (STD (and MW (STD): mean standard OD.

Any sample with an OD more than the upper cut-off was regarded as positive and any sample with an OD less than the lower cut-off was regarded as negative. Moreover, ODs between the lower and upper cut-offs were reported as borderline. In order to report the seroprevalence of Q fever against phases I and II, the positive and borderline results were reported separately. Moreover, to investigate the risk factors, borderline cases were put in the group of negative results.

### Statistical analysis

A 95% confidence interval in Stata version 11 (Stata Corp, College Station, TX, USA) was applied for data analysis. The risk factors for seroprevalence of Q fever were evaluated by logistic regression. In the crude Odds Ratio of the variable of occupations, those referring to laboratories were regarded as the reference group and the percentage of the seroprevalence among other groups was evaluated by drawing a comparison with this reference group.

## Results

In this study, 367 serum samples were taken from five groups, determined as occupations exposed to the risk of Q fever infection, including animal husbandry workers (n = 113), farmers (n = 82), butchers and slaughterhouse workers (n = 61), park rangers (n = 35), and those who referred to the laboratories (n = 76) in three cities of Ilam, Dehloran, and Mehran. Out of all studied cases, 76.29% (n = 280) were male, 89.86% (n = 328) were married. Also, 45.18% (n = 164), 44.08% (n = 160), and 10.74% (n = 39) were living in urban, rural, and nomadic areas, respectively (S1 File). The mean (± SD) age of the tested subjects was 40.54 (±13.55) years old ranging from 18 to 78 years of age.

Furthermore, out of all 367 serum samples studied, 24.38% (88 samples) and 5.54% (20 samples) were positive and borderline, respectively, for phase I. in comparison, 26.37% (96 samples) and 15.38% (56 samples) were positive and borderline, respectively, for phase II. Overall, 32.42% of all samples had a positive titer against Q fever against either phase I or II or both.

The risk of infection with Q fever in Mehran (49.15%) and Dehloran (35.29%) was 4.5 and 2.5 folds higher, respectively, when compared with Ilam (16.78%). As Ilam city is the capital of the Ilam province with a much higher population, its population is less in contact with animals compared with Mehran and Dehloran. In this regard, animal husbandry workers (45.13%) showed a four-fold increase in the risk of Q fever in comparison with those who referred to the laboratories (17.11%le(OR = 3.99,95% CI:1.97–8.05). In comparison, urban citizens (21.88%), rural (35.98%), and nomads (61.54%) showed a higher seroprevalence of the disease.

Moreover, individuals with an age of >50 years showed a 2.6-fold risk of infection increase in compared to those younger than 30 years (OR = 2.58, 95% CI: 1.39–4.79). The risk of positive antibodies against *C*. *burnetii* among uneducated persons and individuals with primary levels of education was significantly higher than those with academic education (OR = 7.78, 95% CI: 3.29–18.39). This data are summarized in Table 1.

**Table 1:** Results from the bivariate logistic regression analysis of demographic predictors using unadjusted odds ratios (ORs).

Variable	Category	Number (% Positive test)	Crude OR (95% CI)	P-value
**Occupation**	Referred to medical Diagnostic Laboratory	76(17.11)	1.00	
** **	Animal husbandry workers	113(45.13)	3.99 (1.97–8.05)	0.012
** **	Butcher and slaughterhouse workers	61(24.59)	1.58 (0.69–3.64)	0.780
	Park ranger	35(22.86)	1.44 (0.53–3.86)	0.580
	Farmer	82(37.80)	2.95 (1.40–6.21)	0.133
**Gender**	Male	280(30.36)	1.00	
** **	Female	87(37.93)	1.40 (0.85–2.32)	0.187
**Age (Year)**	18–30	102(26.47)	1.00	
** **	31–40	100(26.00)	0.98 (0.52–1.83)	0.939
** **	41–50	81(32.10)	1.31 (0.69–2.49)	0.405
** **	>50	81(48.15)	2.58 (1.39–4.79)	0.003
**Area of residence**	Urban	160(21.88)	1.00	
** **	Rural	164(35.98)	2.01 (1.22–3.28)	0.006
** **	Nomad	39(61.54)	5.71(2.71–12.05)	<0.001
**Education**	Academic	63(12.70)	1.00	
	Diploma	94(21.74)	1.91 (0.78–4.66)	0.155
	Secondary	48(22.92)	2.04 (0.75–5.56)	0.162
	Primary	77(47.37)	6.19 (2.60–14.73)	<0.001
** **	Having little or no formal education	81(53.09)	7.78 (3.29–18.39)	<0.001
**County**	The Ilam	143(16.78)	1.00	
	Mehran	120(48.33)	4.64 (2.63–8.17)	<0.001
	Dehloran	103(34.95)	2.66 (1.47–4.84)	0.001

The mean duration of work experience among the study groups was 19.47 (±13.72) years. About 68.51% of respondents considered themselves at risk of exposure to zoonotic diseases. The risk of seropositive cases among those exposed to zoonotic diseases was 2 folds more than others (OR = 1.84, 95% CI: 1.11–3.04).

Among the tested samples, 67.43% reported any type of exposure to blood, secretions, or viscera of animals in their houses or workplaces. The history of exposure to an infected or dead animal and exposure to an animal during delivery was 23.56% and 57.06%, respectively. During the last year of the study, 7% of the participants reported cutting themselves for more than five times. However, the risk of the seropositive status of *C*. *burnetii* did not show any significant difference in any of the mentioned groups.

Out of all the studied samples, 63.81% had a history of hunting or taking the meat of hunted animals. Moreover, 61.22% were keeping domestic animals. The risk of the seropositive status of Q fever among those who were keeping sheep or goats was significantly higher than those who were not keeping these animals (OR = 7.67, 95% CI: 1.75–33.61). The risk of Q fever in those exposed to arthropod bites (37.70%) was higher than that of others (20.54%) (OR = 2.34, 95% CI: 1.39–3.96). These results are presented in Table 2.

**Table 2:** The relationship between behavioral characteristics and Q fever seropositivity in The Ilam Province (2015).

Variable	Number having the variable (%Seropositive)	Number not having the Variable (% Seropositive)	Odds Ratio (95% CI)	P-value
Attitude*	246(36.59)	113(23.89)	1.84 (1.11–3.04)	0.018
**Splashing animal fluids on face/body**	204(35. 29)	99(34.34)	1.04 (0.63–1.73)	0.871
**Exposure to ill or dying animals**	86(29.07)	276(33.70)	0.81 (0.48–1.37)	0.425
**Hunting/consumption** **of wild animal meat**	230(32.17)	129(34.11)	0.92 (0.58–1.45)	0.708
Rabbit	8(50.00)	116(32.76)	2.05 (0.49–8.66)	0.327
Partridge	95(30.53)	29(44.83)	0.54 (0.23–1.27)	0.157
Fox	1(100.00)	123(33.33)	5.96 (0.24–149.6)	0.115
Mongoose	2(100.00)	122(32.79)	10.19 (0.48–217.1)	0.081
Emigrant/feral Birds	44(25.00)	80(38.75)	0.53 (0.23–1.19)	0.124
Mountain animals	89(34.83)	35(31.43)	1.17 (0.51–2.69)	0.719
Others**	3(33.33)	122(34.43)	0.95 (0.08–10.81)	0.969
**Animals Keeping**	219(38.81)	139(23.02)	2.12 (1.31–3.43)	0.002
Cattle	77(37.66)	145(39.31)	0.93 (0.53–1.65)	0.810
Goats and sheep	199(42.21)	23(8.70)	7.67 (1.75–33.61)	0.007
Rabbit	8(37.50)	214(38.79)	0.95 (0.22–4.07)	0.942
Horse / Donkey	60(33.33)	162(40.74)	0.73 (0.39–1.35)	0.315
**Hunting Meat Consumption** **(5 times or more)**	21(28.57)	99(33.33)	0.80 (0.28–2.25)	0.673
**Unpasteurized milk and dairy**	179(37.43)	181(28.18)	1.52 (0.98–2.38)	0.062
Arthropod bite***	252(37.70)	112(20.54)	2.34 (1.39–3.96)	0.001
**Disinfection of hands/face**	75(33.33)	80(36.25)	0.88 (0.45–1.70)	0.703
**Cutting hand/year (5 times or more)**	17(35.29)	227(38.33)	0.88 (0.31–2.46)	0.804

* See themselves as at high risk for zoonotic diseases

** Includes weasel, mongoose, jackal, wild boar, and other wild animals

*** Tick bites, Mosquito bites, and Flea Bites

Total induvial: 367

## Discussion

In this study, the seroprevalence of antibodies against *C*. *burnetii* was investigated in high-risk populations in the Ilam province in the west of Iran. The seroprevalence of IgG antibody against phases I and II of the disease was 24.38 and 26.37%, respectively, with 32.42% as the overall seroprevalence. Previous studies conducted in Iran demonstrated similar results. For example, in a study conducted in Sistan and Baluchistan, in the southeast of Iran, the risk among slaughterhouse workers and butchers was examined and found that the seroprevalence of Q fever was 22.5% in 2016 [17]. In another study in Kurdistan province, west of Iran, the seroprevalence of *C*. *burnetii* was reported 27.83% among hunters and their family members, butchers, the staff of public health centers, and patients who referred to laboratories [15]. Moreover, studies conducted in the neighboring (west and northeast) countries of Iran have also reported high seroprevalence of Q fever. In two studies conducted in Turkey, the seroprevalence of Q fever among high-risk occupations and blood donors was 50.9% and 35.1%, respectively. The highest prevalence rates were detected in abattoir workers, butchers, and farmers (32.8%) [14, 31]. Another study in rural areas of Azerbaijan showed that the seroprevalence of Q fever was 60.2%. Risk factors associated with Q fever seropositivity were age, contact with goats, cats, and rodents, and mosquito bites [32]. Also, the IgG antibody of *C*. *burnetii* was detected in 20% of aborted women in Iraq [33]. However, it seems that the higher seroprevalence of this disease in the Ilam province is related to the economic activities of people who are mainly engaged in holding livestock and farming, which lead to more exposure to livestock. Exposure to domestic animals is considered to be a crucial risk factor in the dissemination of *C*. *burnetii* among human populations [10, 15]. The main route of infection is often inhalation of contaminated dust from infected animal feces, urine, milk and birth products [8]. The most significant animal reservoirs for human infection seems to be associated with farms and include livestock such as cattle, goats, and sheep [10]. The latter populations are believed to be the main reservoirs for urban outbreaks of Q fever [28, 34]. The results of this study, which concur with those published from other studies, have also demonstrated that the risk of Q fever infection in rural and nomadic areas, which have greater exposure to livestock, were 2 and 5.7 folds more than urban areas, respectively [35]. The risk of seropositive cases among animal husbandry workers was 4 folds more than the controls (those who referred to the laboratories). Furthermore, our results also revealed that the risk of seropositive status of Q fever among those who were keeping animals was higher than those who were not keeping any animals (OR = 2.12, 95% CI: 1.31–3.43; p-value = 0.002); and among those who kept sheep or goats had a higher risk (OR = 7.67, 95% CI: 1.75–33.61; p-value = 0.007). Hence, to the best of our knowledge and according to the results of this study, more attention should be paid to a high seroprevalence of Q fever in rural and nomadic areas and occupations that are directly engaged with livestock.

Previous studies have documented the presence of infected ticks in various parts of Iran [36, 37]. In this regard, a study conducted in 1975 reported that around 10% out of 1450 ticks collected from all over the country were infected with *C*. *burnetii* [38]. Moreover, another study in 2009 reported infection with *C*. *burnetii* in 160 ticks collected from domestic livestock (sheep and goats) in Kerman province, the southeast of Iran [39]. Accordingly, a study conducted on Namibian blood donors in 2014 showed that the risk of exposure to *C*. *burnetii* among those who were exposed to domestic animals with recent diagnosis of tick born fever (40%) was 2 folds more than those who were exposed to domestic animals without tick born fever (22.9%) [40]. On the other hand, exposure to tick bite has been regarded as a risk factor in Q fever infection [11]. The results of the present study also showed that the risk of Q fever among those with arthropod bites (tick, flea, and mosquito) was twice more in comparison with those without arthropod bites.

Older ages are factors that significantly are related to an increased risk of Q fever [15, 41, 42]. This study presented that individual 50 years of age and older are at a 2.6-fold increased risk of Q fever compared to others. Yet, another study in Kurdistan also presented that individual older than 50 years are at 2-folds increased risk of Q fever [15]. In an outbreak of Q fever in Switzerland, those individuals older than 15 years of age were at a 5-fold increased risk of Q fever compared with the younger ones [32].

In accordance with the results of other studies, we concluded that lower education is a crucial risk factor regarding the increased risk of Q fever [43]. Hence, it can be deduced that an increase in the level of general knowledge about the disease and its transmission routes among populations and high-risk occupations, in particular, can act as a preventive factor in decreasing the Q fever infection.

In this study, those who referred to the laboratories were taken as the reference group. The infection rate was lower in this group compared to others. However, they might not be a good representative of the general population and their referral to the laboratories may imply the presence of the disease at the time of referral, which would increase the seroprevalence of Q fever among them compared with the real general population. Furthermore, the sample size in different subgroups under study was not the same; consequently, the power of the test in various groups was different. Such a difference in the number of samples somehow originates from differences in the size of these groups and variations in accessing them.

In this study, the analytical cross-sectional survey was used to investigate the association between the putative risk factor and seroprevalence of Q fever disease. However this type of study is limited in its ability to draw valid conclusions as to the association between the risk factors and health outcomes. Since temporality of association is a strong criterion for causality, cross-sectional studies cannot prove causality but help to generate causal hypotheses. It was better to evaluate the risks by cohort studies, but it was not feasible for this study.

Despite all the mentioned limitations, this study, together with some other studies recently conducted in certain provinces of Iran, demonstrated that Iran is an endemic area for Q fever. Hence, it is highly suggested conducting epidemiologic studies in a vaster spectrum in order to evaluate the seroprevalence of the disease in the general population. Moreover, via educational programs for physicians and health personnel, they would become more sensitive about the diagnosis and treatment of this disease. Furthermore, needed information would better be supplied for the general population in respect with the disease and its transmission routes using media. Finally, intersectional collaborations, serological surveillance of imported livestock, and using active policies must be seriously taken into consideration.

## Supporting information

S1 File Raw data from high-risk occupations in the Ilam province. (PDF)
